# Identification and validation of integrated stress-response-related genes as biomarkers for age-related macular degeneration

**DOI:** 10.3389/fmolb.2025.1583237

**Published:** 2025-07-16

**Authors:** Jingyi Niu, Ling Jin, Yijun Hu, Yiting Wang, Xiaoning Hao, Wenwen Geng, Ruirui Ma

**Affiliations:** ^1^ Department of Ophthalmology, The People’s Hospital of Baoan Shenzhen, The Second Affiliated Hospital of Shenzhen University, Shenzhen, China; ^2^ Department of Ophthalmology, Guangdong Provincial People’s Hospital, Guangdong Academy of Medical Sciences, Guangdong Provincial People’s Hospital of Southern Medical University, Guangzhou, China

**Keywords:** age-related macular degeneration, biomarkers, integrated stress response, SLFN11, Grin1

## Abstract

**Background:**

Age-related macular degeneration (AMD) is a prevalent ocular condition associated with aging, serving as a significant contributor to vision loss among middle-aged and older individuals. Studies have shown that AMD and integrated stress response (ISR) are associated with oxidative stress, but no specific molecular mechanisms have been identified. Therefore, this study aimed to identify potential biomarkers for AMD through bioinformatics analysis based on the transcriptome database and integrated stress response related genes (ISR-RGs).

**Methods:**

Transcriptomic data GSE76237, GSE247168, and ISR-RGs were sourced from public databases and related literature. The biomarkers associated with AMD were identified by differentially expressed gene (DEG) analysis, intersection of common DEGs, and ISR-RGs machine algorithm. After that, nomograms, GSEA, and immune infiltration analysis were performed for the biomarkers. The effects of transcription factors (TFs) and miRNAs on biomarkers were then explored by constructing a TF-biomarker–miRNA regulatory network. In addition, potential effective drugs of the biomarkers were explored by constructing a biomarker–effective drug interaction network. Finally, we verified the gene expression of the biomarkers by RT-qPCR.

**Results:**

We obtained 2,567 and 1,454 DEGs in GSE76237 and GSE247168, respectively. The up- and downregulated genes shared in both datasets were intersected with ISR-RGs taken to obtain eight candidate genes. SLFN11 and GRIN1 were identified as common biomarkers for AMD. An analysis of the nomogram model of biomarkers revealed good diagnostic predictive abilities (AUC > 0.7). SLFN11 and GRIN1 were mainly enriched in pathways such as proteasome, lysosome, and neuroactive ligand receptor interaction. In addition, the disease group’s monocyte expression was significantly higher than that of the control group in GSE76237 (p < 0.01). We obtained thirteen relevant miRNAs and 27 TFs by prediction, with three shared TFs, and seventeen potentially effective drugs were predicted. RT-qPCR validation showed in AMD patients, and SLFN11 and GRIN1 expression was significantly higher than controls (p < 0.05). Only SLFN11 expression was consistent with the bioinformatics analysis.

**Conclusion:**

SLFN11 and GRIN1 were identified as AMD biomarkers, exhibiting robust diagnostic performance and providing new insights into the condition.

## 1 Introduction

Age-related macular degeneration (AMD) is a prevalent and irreversible condition that poses a serious threat to vision (Pennington and DeAngelis, 2016). AMD is marked by a gradual deterioration of the central part of the retina, primarily affecting the retinal photoreceptors, the retinal pigment epithelium (RPE), Bruch’s membrane (BM), and the choroidal microcirculation surrounding the macula (Pennington and DeAngelis, 2016). In developed nations, AMD stands as the foremost cause of significant vision impairment in individuals aged 55 and older, contributing to 6%–9% of cases of legal blindness globally (Wong et al., 2014; Jonas et al., 2017). Projections suggest that by 2040, nearly 288 million people around the world will be impacted by AMD (Wong et al., 2014).

AMD is a multifaceted condition, largely due to the interplay of numerous risk factors that contribute to its development. The disease’s origins stem from a combination of demographic influences such as age, gender, and ethnicity with epidemiological factors like body mass index, smoking habits, dietary patterns, and genetic variations, including mutations in the complement cascade. Environmental elements such as prolonged exposure to sunlight and certain chemicals also play a significant role (Chakravarthy et al., 2010; Chakravarthy et al., 2007; Cruickshanks et al., 2001; Tien et al., 2020). Among these, oxidative stress and impaired choroidal blood flow have emerged as central drivers in the progression of AMD (Bellezza, 2018; Lipecz and Miller, 2019; Rakoczy et al., 2002). Oxidative stress, in particular, is a key player in age-related conditions, including AMD, as the retina demands a disproportionately higher amount of oxygen than other tissues in the body (Beatty et al., 2000). A hallmark of AMD is the dysfunction of retinal pigment epithelial cells, which arises from the cumulative impact of aging, genetic predispositions, and environmental exposures. This dysfunction both contributes to and is exacerbated by oxidative stress, creating a vicious cycle that underpins the disease’s progression (Ruan et al., 2021).

Early to intermediate stages of AMD are marked by the presence of lipid–protein accumulations known as drusen, which can be found in the BM. This membrane is a complex structure that encompasses the basement membrane of the retinal RPE (Orozco et al., 2023). It is hard to diagnose and treat during this term as it lacks sensitive biomarkers and effective treatment. The patient is usually advised to live a healthy lifestyle and take supplements with antioxidant vitamins and minerals according to the Age-Related Eye Disease Study 2 (AREDS2) formula (vitamins C and E, lutein, zeaxanthin, zinc oxide, cupric oxide, etc.) in order to prevent the further development of AMD (American Academy of Ophthalmology Preferred Practice Pattern Retina/Vitreous Committee, 2023).

Advanced AMD manifests in two primary forms, both of which result in significant central vision impairment. The “wet” variant involves subretinal neovascularization (NEO) which leads to the deterioration of retinal function, while the “dry” form, known as geographic atrophy (GA), is marked by the irregular degeneration of the RPE and photoreceptors. Interestingly, these advanced stages can coexist in a single patient, suggesting that the underlying progression pathways are not mutually exclusive (Owen et al., 2019). While anti-VEGF (anti-vascular endothelial growth factor) therapies have proven effective in managing wet AMD, treatment options for the dry form remain limited, highlighting a critical gap in current therapeutic strategies (Orozco et al., 2023).

To date, a staggering 90% of individuals worldwide suffering from AMD remain without viable treatment options (Ambati and Fowler, 2012). This pressing reality underscores the critical need to deepen our understanding of the disease’s underlying mechanisms, identify reliable biomarkers, and develop precise, targeted therapies to address this growing health challenge.

Integrated stress response (ISR) plays a key role in maintaining cell homeostasis as it is the core regulatory mechanism for cells in coping with various internal and external stressors (Paternoga et al., 2025). ISR is also a research hotspot partly because it relates to many important diseases, including metabolic diseases (Delepine et al., 2000; Harding et al., 2001; Zhang et al., 2002; Senee et al., 2004), pulmonary diseases (Emanuelli et al., 2020), diseases of the nervous system (Lin et al., 2014; Vasudevan et al., 2022), cancer (Dey et al., 2015; Nguyen et al., 2018; Verginadis et al., 2022), Alzheimer’s disease (Ma et al., 2013; Yang et al., 2016), peripheral neuropathies (Pennuto et al., 2008; Antonio et al., 2013; Spaulding et al., 2021; Amila et al., 2021), and Down’s syndrome due to chromosomal trisomy (Zhu et al., 2019). The core regulatory pathway of ISR revolves around four kinases (PERK, GCN2, PKR, and HRI) (Pakos-Zebrucka et al., 2016) which respond to different stress signals. PERK is sensitive to endoplasmic reticulum stress. GCN2 mainly responds to amino acid starvation. PKR and HRI are activated by viral infection and heme deficiency, respectively (Peichuan et al., 2002a; Peichuan et al., 2002b; Williams, 1999; Wang et al., 2013). Once activated, they reprogram protein synthesis by phosphorylating eukaryotic translation initiation factor 2α (P-eIF2α). The formation of P-eIF2α inhibits global protein translation while selectively initiating the translation of specific mRNAs such as ATF4 (Wek, 2018). ATF4 can further activate downstream stress adaptation genes (such as antioxidant and metabolic regulatory genes), promote cell repair, or determine apoptosis according to stress intensity (Harding et al., 2003). Therefore, understanding the mechanism of ISR can better explore its role in the occurrence and development of diseases and provide new ideas and targets for the treatment of related diseases.

The stress response protein “regulated in development and DNA damage 1” (REDD1), sometimes referred to as “RTP801” or “DDIT4,” has been identified as a contributing factor in the onset of oxidative stress (Shoshani et al., 2002; Miller et al., 2022; Miller et al., 2021; Miller et al., 2020). In their recent investigation, S. M. Subrahmanian and colleagues examined how REDD1 influences damage to retinal pigment epithelium (RPE), retinal degeneration, and its possible role in the pathogenesis of AMD. They achieved this by administering the oxidizing agent sodium iodate (NaIO3) to mice, which simulates dry AMD by causing RPE dysfunction and an increase in macrophage presence. This ultimately leads to photoreceptor damage, thinning of both the outer and inner segments, and diminished visual acuity (Shoshani et al., 2002; Miller et al., 2022; Miller et al., 2021; Miller et al., 2020). Their results indicate that REDD1 levels increased in the retinas of mice treated with NaIO3 and, notably, that the removal of REDD1 was enough to avert oxidative stress, the activation of immune cells, and alterations in retinal structure (Espitia-Arias et al., 2023; Moriguchi et al., 2018; Yang et al., 2021). These findings imply that REDD1 may significantly contribute to the damage of RPE and photoreceptors associated with dry AMD (Subrahmanian et al., 2024). ISR and AMD thus have a close relationship, but the mechanism is unknown.

This study explores the biomarkers of ISR relating to AMD through the analysis and techniques of diverse bioinformatics across public databases. In addition, the signaling pathways associated with these biomarkers and their correlation with miRNAs, TFs, and potentially effective drugs were investigated to provide valuable insights for ophthalmologists in the diagnosis and treatment of AMD patients.

## 2 Materials and methods

### 2.1 Data source

In this study, AMD-related datasets were collected from the Gene Expression Omnibus (GEO) database (https://www.ncbi.nlm.nih.gov/geo/). GSE76237, leveraging the GPL6244 platform, consisted of peripheral blood mononuclear cell (PBMC) samples from 14 AMD patients and 15 unaffected control subjects. GSE247168, which was based on the GPL29480 platform, included PBMC samples from nine AMD patients and seven healthy control subjects. Meanwhile, 1,797 integrated stress-response-related genes (ISR-RGs) were extracted and integrated from five studies (Fukuoka et al., 2022; Costa-Mattioli and Walter, 2020; Wang and Li, 2023; Deardorff et al., 1986; Chen et al., 2024).

### 2.2 Differential expression analysis

Differences in gene expression levels between AMD samples and control samples in GSE76237 were compared using the R limma package (v 3.54.0), with the screening conditions |log_2_fold change (FC) | > 0 and p < 0.05 to screen differentially expressed genes (DEGs). In addition, differences in gene expression levels between AMD samples and control samples in GSE247168 were compared using the DESeq2 package (v 1.38.3) with the same screening conditions for screening DEGs. The intersection of up- and downregulated genes in DEGs of GSE76237 and GSE247168 were taken separately to obtain the shared up- and downregulated genes, which were termed “common upregulated DEGs” and “common downregulated DEGs,” respectively. The volcano plot and heatmap for DEGs were drawn by the ggplot2 package (v 3.4.4) and the Pheatmap package (v 2.14.0).

### 2.3 Enrichment analysis of candidate genes

Further analysis involved taking the intersection of common upregulated DEGs, common downregulated DEGs, and ISR-RGs to gain candidate genes through the VennDiagram package (v 1.7.3). Subsequently, the clusterProfiler package (v 4.7.1.003) was used to conduct enrichment analyses for Gene Ontology (GO) and the Kyoto Encyclopedia of Genes and Genomes (KEGG) on the candidate genes (p Adjust Method = “BH,” p-value cut off = 0.05, FDR < 0.05). The results were visualized with ggplot2 package (v 3.4.4).

### 2.4 Recognition of biomarkers for AMD

First, based on the candidate genes, the feature genes were obtained by least absolute shrinkage and selection operator (LASSO) using the glmnet package (v 4.1-4) in GSE76237. The most strongly associated features were selected when lambda reached its minimum value, yielding the lowest error rate and identifying LASSO-GSE76237. Meanwhile, LASSO-GSE247168 were obtained by LASSO in GSE247168. The common LASSO-feature genes were obtained by taking the intersection of LASSO-GSE76237 and LASSO-GSE247168. Subsequently, with the support of the e1071 package (v 4.1-4), candidate genes were incorporated into the support vector machine-recursive feature elimination (SVM-RFE) algorithm across GSE76237 and GSE247168 individually. For each feature, the error rate was calculated using five-fold cross-validation. The feature genes selected from both datasets were defined as “SVM-RFE-GSE76237” and “SVM-RFE-GSE247168,” respectively. The common SVM-RFE-feature genes were obtained by taking the intersection of SVM-RFE-GSE76237 and SVM-RFE-GSE247168. Biomarkers were identified by overlapping LASSO- and SVM-RFE-feature genes.

### 2.5 Construction and evaluation of nomogram

To assess the capacity of biomarkers to differentiate between diseased and normal samples in GSE76237 and GSE247168, two distinct nomograms were constructed with the rms package (v 6.5.0) tailored to enhance the diagnostic accuracy for AMD. In each nomogram, biomarkers were represented by line segments scaled to indicate possible scoring ranges, with the segment length depicting the biomarker’s contribution to the predicted outcome. Points on these segments denoted the individual scores for each biomarker, while the aggregate of these points—“total points” —was employed to evaluate the disease risk associated with each profile, where a higher total score suggested a higher risk of disease. Further analyses were performed to validate the nomogram’s performance, notably by plotting the calibration curve to assess its predictive power. The pROC package (v 1.18.0) was also utilized to construct a receiver operating characteristic (ROC) curve, thereby evaluating the nomogram’s diagnostic effectiveness through area under the curve (AUC) metrics. In addition, decision curve analysis (DCA) (rmda package, v 1.6, http://mdbrown.github.io/rmda/) was executed to ascertain the clinical utility of the nomogram by measuring its net benefit.

### 2.6 Functional analysis of biomarkers

To explore the functional enrichment of biomarkers adopting the clusterProfiler package, gene set enrichment analysis (GSEA) was performed on biomarkers in GSE76237 and GSE247168. In detail, Spearman correlation analysis using the “corrplot R” package was conducted to calculate correlation coefficients between biomarkers and other genes in each dataset. Genes were ranked based on these coefficients, and each biomarker was associated with a list of correlated genes. The “msigdbr” package (v 7.5.1) was used to download “c2. kegg.v7.4. symbols.gmt” as the background gene set. GSEA was then performed on ranked genes within the background gene set. Pathways with a p.adjust < 0.05 were considered significantly enriched and visualized for the top-five pathways per gene operating the enrISplot package (v 1.18.4).

### 2.7 Immune infiltration analysis

Considering the number of samples, GSE76237 was chosen to study the infiltration abundance of immune cells in AMD and normal individuals. Utilizing the CIBERSORT algorithm (v 0.1.0), the infiltration abundance of 22 types of immune cells in these samples were calculated. The differences in these immune cells between AMD and control samples were then compared (p < 0.05).The psych package (v 2.2.5) was used to perform Spearman correlation analysis to assess the relationship between these differential immune cells and biomarkers in GSE76237. This analysis helped identify which types of immune cells were associated with specific biomarkers.

### 2.8 Construction of regulatory network

The construction of regulatory networks was helpful for uncovering the molecular regulatory mechanisms of biomarkers, thereby providing new insights for AMD research. Initially, potential microRNAs (miRNAs) regulating these biomarkers were predicted through the miRDB database (http://www.mirdb.org/). In the following, the chip-seq data of the ENCODE database (https://www.encodeproject.org/) was used to predict transcription factors that regulate biomarkers online using NetworkAnalyst (https://www.networkanalyst.ca/). Afterward, the regulatory networks involving miRNA-mRNA, TF-mRNA, and TF-mRNA-miRNA were visualized using Cytoscape software.

### 2.9 Drug prediction

The identification of biomarker-associated targeted drugs was conducted by utilizing the Drug Signatures Database (DSigDB) (https://dsigdb.tanlab.org/DSigDBv1.0/). A network diagram of targeted drugs and biomarkers was generated via Cytoscape software, facilitating the exploration of potential therapeutic agents for treating AMD.

### 2.10 Expression analysis of biomarkers

To confirm the expression levels of biomarkers in both AMD and control groups, the study examined these levels across both the training and validation sets. Further validation was carried out using reverse transcription–quantitative polymerase chain reaction (RT-qPCR). This research utilized 11 peripheral blood mononuclear cell samples obtained from patients at The People’s Hospital of Baoan Shenzhen, comprising five samples from AMD patients and six from control subjects. The study received ethical approval from the Ethics Committee of The People’s Hospital of Baoan Shenzhen, and all participants provided written informed consent. To assess biomarker expression, total RNA was extracted from the samples using TRIzol (Ambion, Austin, United States) in accordance with the manufacturer’s protocol. The first strand of complementary DNA (cDNA) was synthesized from 2 μg of total RNA using the SweScript First Strand cDNA Synthesis Kit (Servicebio, Wuhan, China), following the guidelines provided. RT-qPCR was performed using the 2×Universal Blue SYBR Green qPCR Master Mix (Servicebio, Wuhan, China), with the reaction program set to 1 min at 95°C followed by 40 cycles of 20 s at 95°C, 20 s at 55°C, and 30 s at 72°C. Primer sequences are detailed in Supplementary Table S1. GAPDH served as the internal reference gene, and gene expression levels were quantified using the 2^−ΔΔCT^ method (PMID: 11846609). The resulting data were visualized using GraphPad Prism 5 (GraphPad Software Inc., United States).

### 2.11 Statistical analysis

All analyses were performed using R software (v 4.2.2). The limma package (v 3.54.0) and DESeq2 package (v 1.38.3) in R language were used for differential expression analysis. The screening criteria were set as |log_2_fold change (FC)| > 0 and p < 0.05. The clusterProfiler package (v 4.7.1.003) was used to conduct GO and KEGG enrichment analyses on the candidate genes. The glmnet package (v 4.1-4) was used for the LASSO regression analysis. The e1071 package (v 4.1-4) was utilized to perform the SVM-RFE algorithm. The rms package (v 6.5.0) was applied to construct nomograms. The pROC package (v 1.18.0) was used to construct the ROC curve. The clusterProfiler package was employed to conduct GSEA on the biomarkers. The CIBERSORT algorithm (v 0.1.0) was used to calculate the infiltration abundance of 22 types of immune cells in the samples. The Wilcoxon test was used to compare the differences in the infiltration abundance of immune cells between AMD samples and control samples, and p < 0.05 indicated that the differences were statistically significant. The psych package (v 2.2.5) was used for Spearman correlation analysis. When validating the biomarkers by RT-qPCR, the 2^−ΔΔCt^ method was used to quantitatively analyze the gene expression levels. P < 0.05 was considered to be significantly different.

## 3 Results

### 3.1 Identification of common upregulated and downregulated DEGs

**FIGURE 1:**
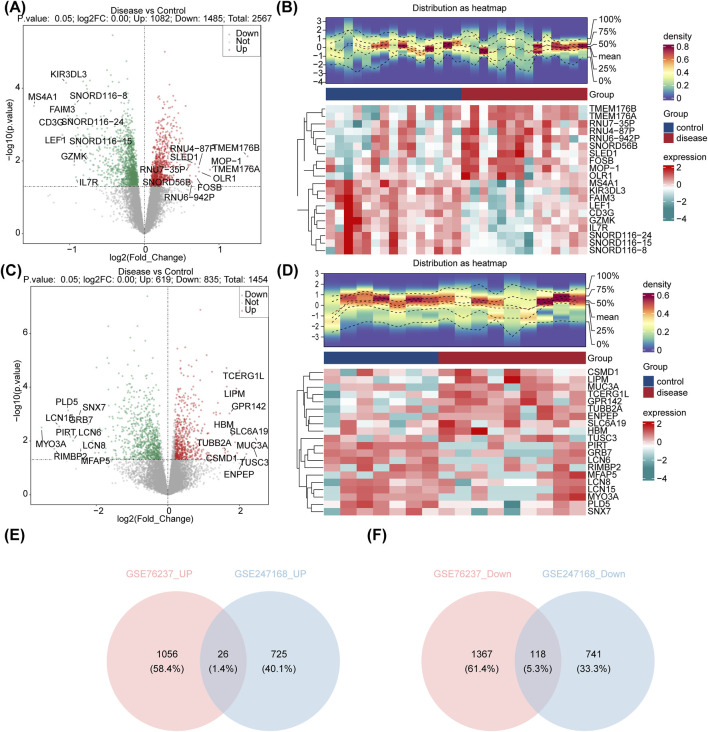
Identification of the DEGs in AMD. **(A)** Volcano plot showing the expression of DEGs between AMD patients and control samples in GSE76237. **(B)** Heatmap showing the top-20 regulated DEGs in GSE76237. **(C)** Volcano plot showing the expression of DEGs between AMD patients and control samples in GSE247168. **(D)** Heatmap showing the top-20 regulated DEGs in GSE247168. **(E)** Venn diagram identifying the common upregulated genes in GSE76237 and GSE247168. **(F)** Venn diagram identifying the common downregulated genes in GSE76237 and GSE247168.

Differential expression analysis revealed 2,567 DEGs between AMD and control samples in GSE76237, with 1,082 upregulated and 1,485 downregulated genes in AMD (Figures 1A,B). Similarly, in GSE247168, 1,454 DEGs were identified between AMD and control samples, with 619 upregulated and 835 downregulated genes in AMD (Figures 1C,D). After crossing, a total of 26 common upregulated DEGs and 118 common downregulated DEGs that were generated (Figures 1E,F).

### 3.2 Exploring the signaling pathways enriched in candidate genes

**FIGURE 2:**
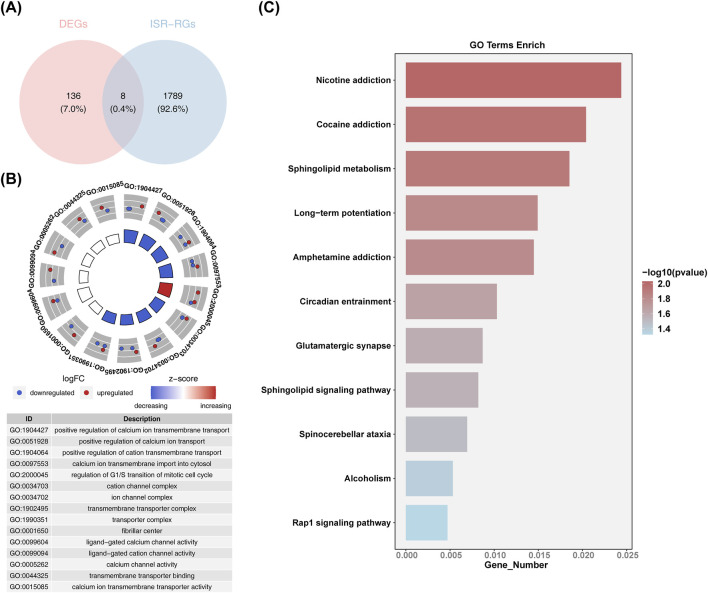
Functional enrichment analysis of candidate genes. **(A)** Venn diagram identifying the candidate genes of common upregulated and downregulated DEGs and 1,797 ISR-RGs. **(B)** Circle diagram of GO enrichment analysis (candidate genes functionally enriched categories in BP, CC, and MF analysis). **(C)** KEGG enrichment analysis: vertical axis is the specific name of the KEGG function, horizontal axis is the number of differential genes released to the corresponding function.

The subsequent intersection of 26 common upregulated DEGs, 118 common downregulated DEGs, and 1,797 ISR-RGs yielded eight candidate genes related to integrated stress response in AMD (Figure 2A): RIC3, GRIN1, AKAP6, SGPP2, STOX1, SLFN11, FAM111A, and PKD2. These were further included in functional enrichment analysis, revealing 352 GO terms encompassing 277 BP, 28 CC, and 47 MF. Notable terms included “positive regulation of cation transmembrane transport,” “transmembrane transporter complex,” “ligand-gated calcium channel activity,” and “calcium channel activity” (Figure 2B). In addition, 11 KEGG pathways were identified, including “nicotine addiction,” “cocaine addiction,” “sphingolipid metabolism,” “long-term potentiation,” and “amphetamine addiction” (Figure 2C). These findings validated the accuracy of the differential analysis and highlight the significance of these pathways in the pathogenesis of AMD.

### 3.3 Identifying SLFN11 and GRIN1 as biomarkers for AMD

**FIGURE 3:**
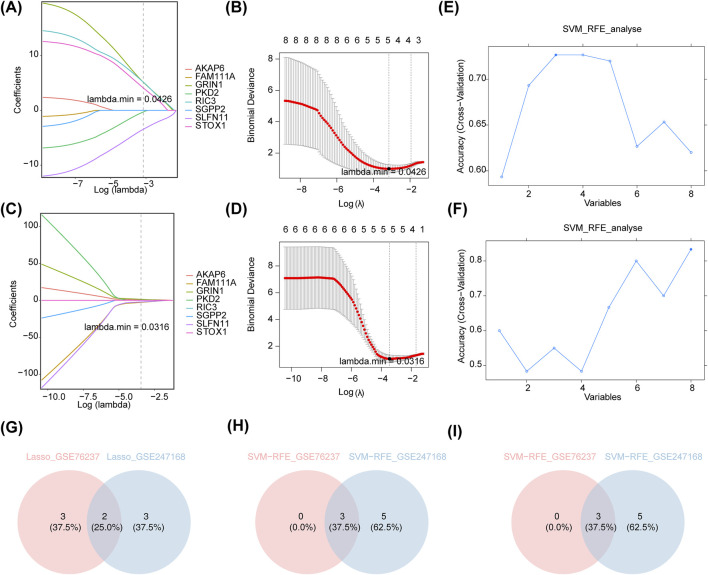
Machine algorithms for signature genes. **(A)** *λ*-curve in lasso regression analysis (distribution of LASSO coefficients for eight genes in GSE76237). Penalty plot of LASSO model with error bars denoting standard errors. The LASSO plot shows that the variations in the size of the coefficients for the parameters decreased as the value of the k penalty increased. **(B)** Log(*λ*)-curve in lasso regression analysis in GSE76237. (Results of cross-validation. Values in the middle of the two dashed lines are the range of positive and negative standard deviations of log(λ). The dashed line on the left indicates the value of the harmonic parameter log(λ) when the model error is minimal). **(C)** *λ*-curve in lasso regression analysis (distribution of LASSO coefficients for eight genes in GSE247168). **(D)** Log(*λ*)-curve in lasso regression analysis in GSE247168. **(E)** Performance of the feature subset selected by SVM on the dataset and the value of the horizontal coordinate corresponding to the highest point of the curve indicates the best genes in GSE76237. **(F)** The performance of the feature subset selected by SVM on the dataset and the value of the horizontal coordinate corresponding to the highest point of the curve indicates the best genes in GSE247168. **(G)** Intersection of LASSO feature genes of the two datasets. **(H)** Intersection of SVM-RFE feature genes of the two datasets. **(I)** Intersection of the results of **(G,H)**.

The eight candidate genes were further refined through LASSO and SVM-RFE for selection. Specifically, in GSE76237, the lowest error rate was achieved when lambda. min was set to 0.0426, resulting in the identification of five LASSO-feature genes 1: SLFN11, PKD2, GRIN1, RIC3, and STOX1 (Figures 3A,B). In GSE247168, the lowest error rate was achieved when lambda. min was set to 0.0316, resulting in the identification of five LASSO-feature genes 2: SGPP2, GRIN1, FAM111A, AKAP6, and SLFN11 (Figures 3C,D). In GSE76237, the highest point of accuracy was selected for the best gene combination SVM-RFE-1, including RIC3, SLFN11, and GRIN1 (Figure 3E). GSE247168 was selected for the highest point of accuracy for the best gene combination SVM-RFE-2, including AKAP6, GRIN1, PKD2, RIC3, SGPP2, FAM111A, SLFN11, and STOX1 (Figure 3F). The intersection of LASSO and SVM-RFE feature genes of the two datasets are taken separately (Figures 3G,H), and finally the intersection of the results of these two machine algorithms’ learning is taken to obtain the AMD core genes SLFN11 and GRIN1 (Figure 3I).

### 3.4 Nomograms of the biomarkers SLFN11 and GRIN1

**FIGURE 4:**
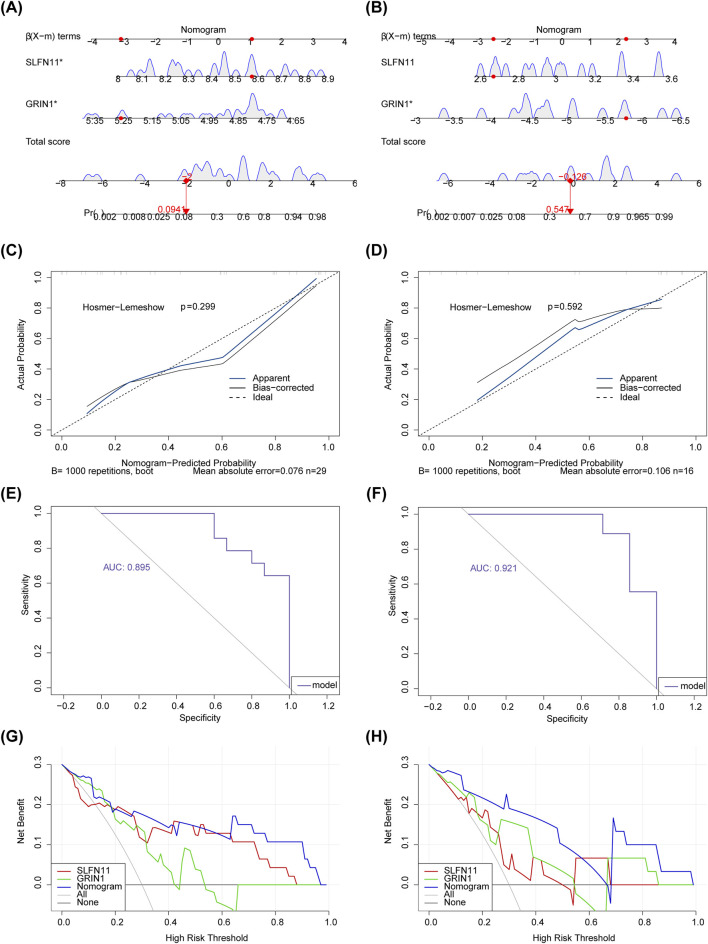
Nomograms of the biomarkers SLFN11 and GRIN1. **(A)** Nomograms of biomarkers SLFN11 and GRIN1 in GSE76237. **(B)** Nomograms of biomarkers SLFN11 and GRIN1 in GSE247168. **(C)** The calibration curve for nomogram in GSE76237. **(D)** The calibration curve for nomogram in GSE247168. **(E)** ROC curve analysis showing the predictive performance of two columnar graphical models in GSE76237. **(F)** ROC curve analysis showing the predictive performance of two columnar graphical models in GSE247168. **(G)** DCA decision curve showing the strong diagnostic prediction ability of the model in GSE76237. **(H)** DCA decision curve showing the strong diagnostic prediction ability of the model in GSE247168.

To further explore the diagnostic predictive ability of biomarkers in AMD, nomograms of biomarkers SLFN11 and GRIN1 were constructed in GSE76237 and GSE247168, respectively (Figures 4A,B). We performed calibration curves for the models separately (Figures 4C,D), and the results showed that the predictive performances of the two columnar graphical models were good (p > 0.05). The fit of the models was evaluated using ROC curves, and the AUCs (Figures 4E,F) in GSE76237 and GSE247168 were 0.895 and 0.921 (AUC > 0.7), respectively, demonstrating that the constructed nomograms could predict the survival of patients well. DCA results further proved that the model has strong diagnostic prediction ability (Figures 4G,H).

### 3.5 Functional analysis of SLFN11 and GRIN1

**FIGURE 5:**
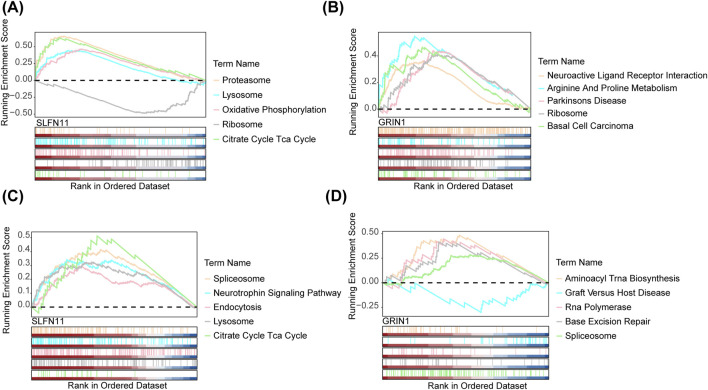
GSEA of the signature genes in AMD. **(A)** GSEA of SLFN11 in AMD by KEGG analysis and GO analysis in GSE76237. **(B)** GSEA of GRIN1 in AMD by KEGG analysis and GO analysis in GSE76237. **(C)** GSEA of SLFN11 in AMD by KEGG analysis and GO analysis in GSE247168. **(D)** GSEA of GRIN1 in AMD by KEGG analysis and GO analysis in GSE247168.

In order to explore the functional enrichment of biomarkers in the dataset, we performed GSEA enrichment analyses of biomarkers in both datasets. In GSE76237, the top-five pathways significantly enriched for SLFN11 were proteasome, lysosome, oxidative phosphorylation, ribosome, and the tricarboxylic acid (TCA) cycle (Figure 5A). The top-five pathways significantly enriched for GRIN1 were neuroactive ligand–receptor interaction, arginine and proline metabolism, Parkinson’s disease, ribosome, and basal cell carcinoma (Figure 5B). In GSE247168, the top-five pathways significantly enriched for the SLFN11 gene were spliceosome, the neurotrophin signaling pathway, endocytosis, lysosome, and the tricarboxylic acid (TCA) cycle (Figure 5C). GRIN1 genes were significantly enriched in the top-five pathways: aminoacyl tRNA biosynthesis, graft versus host disease, RNA polymerase, base excision repair, and spliceosome (Figure 5D). Overall, the two datasets were each enriched for a number of shared pathways involved in multiple cellular functions and physiological activities.

### 3.6 Correlation between biomarkers and immune cells

**FIGURE 6:**
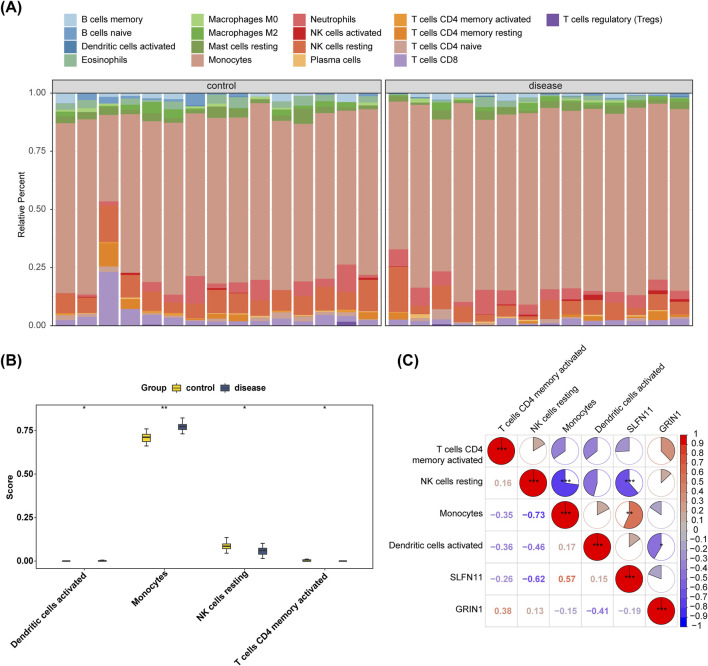
Infiltration levels of 22 immune cells were analyzed between the AMD and control groups in GSE76237. **(A)** Bar-plot displaying the relative proportion levels of all immune cells. **(B)** Differential analysis of four immune cells’ infiltration between AMD and control groups in GSE76237. **(C)** Spearman correlation analysis of biomarkers and four immune cells in GSE76237.

Understanding the correlation between biomarkers and immune cells will help us further explore the mechanism of action of the disease. Figure 6A shows the relative proportions of 22 immune cells between the AMD and control groups in GSE76237. The proportional difference in the infiltration abundance of 22 immune cell species between AMD samples and control group samples in GSE76237 was analyzed. Four types of cells—activated dendritic cells, monocytes, resting natural killer (NK) cells, and activated memory CD4 T cells—were significantly different between the two groups, with monocytes being more highly expressed in the disease group (p < 0.01) (Figure 6B). Ultimately, we conducted a Spearman correlation analysis of biomarkers and differential immune cells which showed that in GSE76237, NK-cell resting exhibited the most significant negative correlation with monocytes at −0.73 (p < 0.001). SLFN11 was positively correlated with monocytes with a correlation coefficient of 0.57 (p < 0.01). GRIN1 was negatively correlated with activated dendritic cells with a negative correlation coefficient of −0.41 (p < 0.05) (Figure 6C).

### 3.7 Establishment of TF–miRNA–mRNA regulatory networks

**FIGURE 7:**
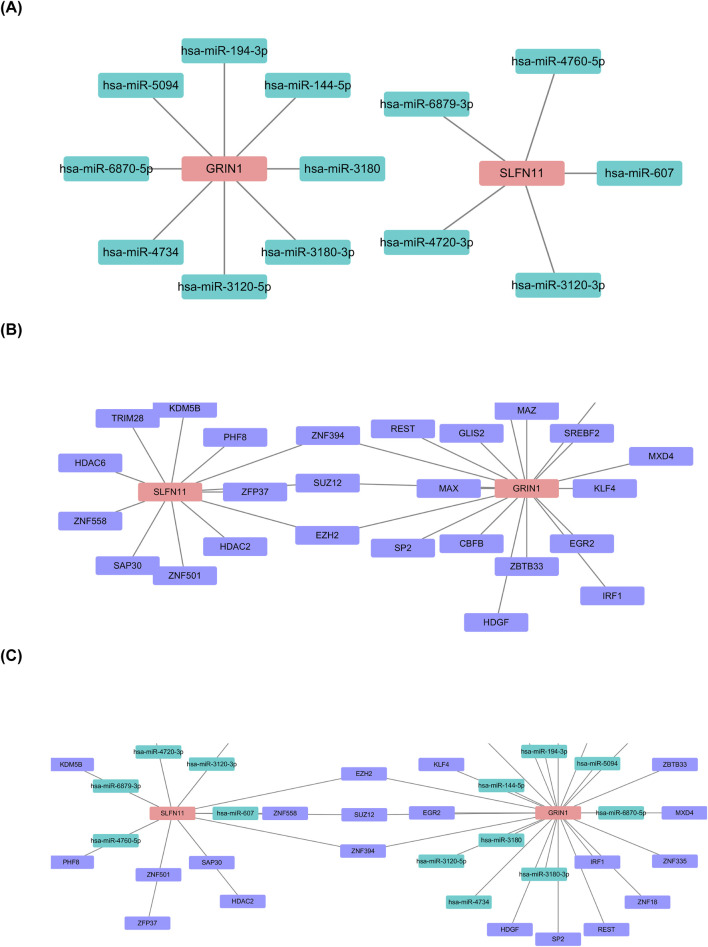
Establishment of TF–miRNA–mRNA regulatory networks. **(A)** Prediction of biomarkers targeting miRNAs. **(B)** Prediction of biomarkers targeting TF showing 29 nodes, 30 edges, and 3 shared TFs (SUZ12, EZH2, and ZNF394). **(C)** TF–miRNA–mRNA regulatory networks showing 35 nodes, 37 edges, 3 shared nodes (SUZ12, EZH2, and ZNF394).

The prediction of biomarkers targeting miRNAs showed 15 nodes, 13 edges, with eight miRNAs predicted for GRIN1 and five for SLFN11 (Figure 7A). Predicted miRNAs included miR-144-5p, miR-607, and miR-194-3p. Prediction of biomarkers targeting TF showed 29 nodes, 30 edges, and 3 shared TFs (SUZ12, EZH2, and ZNF394) (Figure 7B). TF–miRNA–mRNA regulatory networks showed 35 nodes, 37 edges, and 3 shared nodes (SUZ12, EZH2, and ZNF394) (Figure 7C).

### 3.8 Prediction of potentially effective drugs by biomarkers

**FIGURE 8:**
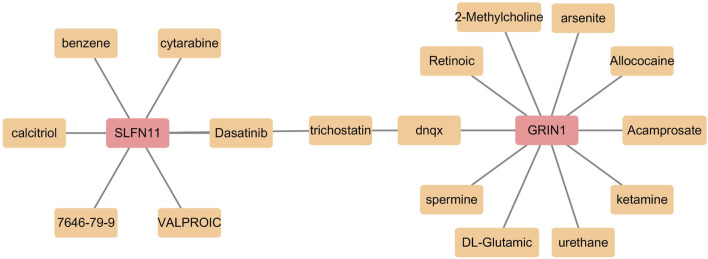
Prediction of potentially effective drugs by biomarkers.

The biomarker-related potential therapeutic drug prediction results showed a total of 19 nodes, 18 edges, and 1 shared node. SLFN11 and GRIN1 shared the drug trichostatin. Eleven drugs were predicted for GRIN1, and seven were predicted for SLFN11 (Figure 8).

### 3.9 RT-qPCR validation of biomarker expression

**FIGURE 9:**
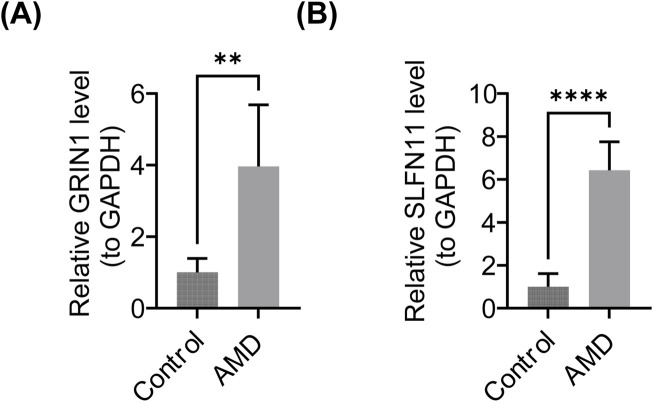
Results of PCR. **(A)** Expression of GRIN1 in the AMD and control groups. **(B)** Expression of SLFN11 in the AMD and control groups.

In this study, the expression levels of the biomarkers were further validated. The expression levels of GRIN1 and SLFN11 were significantly upregulated in the AMD group (p < 0.05) (Figures 9A,B). SLFN11 expression was consistent with the bioinformatics analysis, while GRIN1 was opposite to it.

## 4 Discussion

AMD impacts millions across the globe as a prominent cause of vision loss (Klein et al., 2007). It can be viewed as a breakdown in the retina’s usual homeostatic processes, where age-related changes, chronic inflammation, lipid and lipoprotein accumulation, oxidative stress, and deteriorated extracellular matrix (ECM) maintenance create a state of imbalance that leads to the disease (Miller, 2013). ISR is a highly conserved signaling pathway that activates in response to stressors, generally downregulating protein synthesis while selectively promoting the translation of specific proteins, including transcription factors (Kalinin et al., 2023). This pathway comes into play under various stress conditions, such as oxidative stress, lack of amino acids, and endoplasmic reticulum stress, finely tuning the cell’s translation and expression profiles (Wek et al., 2023). Damage from oxidative stress to the retinal pigment epithelium (RPE) is regarded as the main pathological factor behind AMD. Nonetheless, the precise role of ISR in the development of AMD remains elusive. Therefore, this study seeks to investigate potential biomarkers related to ISR-related genes (ISR-RGs) in AMD and aims to offer new insights for its early diagnosis and treatment.

We obtained 2,567 DEGs in GSE76237 and 1,454 DEGs in GSE247168. The up- and downregulated genes shared in both datasets were intersected with ISR-RGs taken to obtain eight candidate genes. Subsequently, SLFN11 and GRIN1 were identified as common biomarkers for AMD.

The Schlafen (SLFN) family of proteins is integral to regulating key biological processes in mammals, including curbing viral replication and modulating immune responses (Mavrommatis et al., 2013; Bustos et al., 2009; Schwarz et al., 1998). Among these, SLFN11, a prominent member of the human Schlafen family, has been identified as a potent inhibitor of human immunodeficiency virus 1 (HIV-1) by leveraging codon usage patterns (Li et al., 2012). Functioning as a putative DNA/RNA helicase, SLFN11 is drawn to stressed replication forks, where it irreversibly halts replication and triggers cell death. This mechanism has positioned SLFN11 as a promising biomarker for predicting sensitivity to cytotoxic chemotherapies, particularly DNA-damaging agents (DDAs) such as topoisomerase I and II inhibitors (e.g., irinotecan and etoposide), DNA synthesis blockers (e.g., gemcitabine), and DNA cross-linking or alkylating agents (e.g., cisplatin) (Zoppoli et al., 2012; Nogales et al., 2015). Additionally, SLFN11 has recently been linked to responsiveness to poly (ADP-ribose) polymerase (PARP) inhibitors (Murai et al., 2016; Lok et al., 2016; Stewart et al., 2017; van Erp et al., 2020; Pietanza et al., 2018). Beyond its antiviral capabilities, emerging research highlights SLFN11’s role in enhancing cancer cell sensitivity to DDAs, making it a potential biomarker for predicting treatment outcomes in ovarian, lung, and colorectal cancers (Zoppoli et al., 2012; Gardner et al., 2017; Deng et al., 2015). Furthermore, SLFN11 has been shown to disrupt stressed replication forks, driving cell death in response to DNA damage (Murai et al., 2018). Most intriguingly, recent findings suggest SLFN11’s involvement in immune regulation, particularly in promoting T-cell infiltration and activation in breast cancer, underscoring its multifaceted role in both cancer therapy and immune modulation (Isnaldi et al., 2019). This study for the first time identified the SLFN11 gene as one of the biomarkers of AMD, but it is worth noting that the specific mechanism of the SLFN11 gene in AMD is rarely reported, and further studies are needed for clarification.

The GRIN gene encodes NMDA receptors, which play a vital role in brain maturation and cognitive abilities. Genetic research has identified a link between *de novo* mutations in GRIN genes and neurological conditions (O'Roak et al., 2011; de Ligt et al., 2013). Pathogenic variants of GRIN genes cause a group of rare genetic neurodevelopmental disorders (Lemke, 2020; Robertson and Baron-Cohen, 2017; Platzer et al., 2019; Benke et al., 2021). Natural variations in the GRIN1 gene, responsible for encoding the essential GluN1 subunit of the NMDA receptor, are linked to significant neurological disorders (Lotten et al., 2023). Pathogenic GRIN1 variants cause intellectual disability (100% of patients), muscular hypotonia (66% of patients), epilepsy (65% of patients), motor dysfunction (48% of patients), cortical visual impairment (CVI; 34% of patients), autism spectrum disorder (ASD; 22% of patients), and sleep problems (15% of patients) (Benke et al., 2021).

Cortical visual impairment (CVI) is a common symptom and sensory characteristic associated with GRIN disorders. (Lipina et al., 2022) observed in approximately one-third of individuals carrying the GluN1 gene, which is why ophthalmologic evaluations are advised for patients with GRIN1 (Platzer et al., 2019). While CVI stems from damage to the brain regions responsible for visual processing, the influence of GRIN1 on retinal function should not be overlooked. In fact, retinal Müller glial cells, which contain the GluN1 subunit, may facilitate the growth of retinal progenitor cells, regulate the expression of glutamate transporters, and support the survival of ganglion cells (Traynelis et al., 2010; Ramírez and Lamas, 2009; Furuya et al., 2012). These processes can significantly impact both the structure and function of the retina.

GluN1 knockdown mice (GluN1KD) serve as a model for GRIN disorders, exhibiting significant alterations in learning, memory, and emotional responses alongside visual deficits stemming from a lack of NMDA receptors (Lipina et al., 2022). Research conducted by Tatiana Lipina and colleagues revealed that these mice experience destabilization in the outer segment of the retina, as well as a reduced quantity and size of Meissner corpuscles—mechanoreceptors located in the hind paw (Lipina et al., 2022).

Furthermore, research has identified new links between the polymorphic loci in the GRIN1 gene (rs6293) and external eating behaviors in individuals with type 2 diabetes, as well as associations of the GRIK3 (rs534131) and GRIA1 (rs2195450) genes with diabetic retinopathy (Kochetova et al., 2020). Notably, glioma patients exhibiting reduced levels of GRIN1 expression tend to have poorer prognoses than those with higher levels of expression (Yang et al., 2021b). Our investigation reveals for the first time that GRIN1 serves as a potential biomarker for AMD, warranting further exploration of its specific mechanisms.

In order to reveal the functions of SLFN11 and GRIN1, we performed GSEA enrichment analyses of the two genes both in GSE76237 and GSE247168, finding that the two datasets were each enriched for a number of shared pathways involved in multiple cellular functions and physiological activities. This indicates that SLFN11 and GRIN1 participate in AMD dependence on multiple, not single, molecular mechanisms. The common pathways significantly enriched for SLFN11 were ribosome and tricarboxylic acid (TCA) cycle in both datasets; there were no common pathways significantly enriched for GRIN1 in both datasets. In GSE76237, the common pathway significantly enriched for SLFN11 and GRIN1 was ribosome. In GSE247168, the common pathway significantly enriched for the SLFN11 and GRIN1 genes was spliceosome. Regardless, all these signaling pathways referring to SLFN11 and GRIN1 were related to most important physiological and pathological processes.

To investigate the relationship between biomarkers and immune cells, we employed immune infiltration analysis to shed light on the underlying mechanisms of AMD. Our findings revealed that in the GSE76237 dataset, the expression of monocytes in the AMD group was notably elevated compared to the control group. Monocytes, as key players in the innate immune system, have previously been shown to serve as a reliable and cost-effective biomarker for identifying patients at high risk of acute exacerbation of chronic obstructive pulmonary disease (AECOPD). Specifically, studies indicate that individuals with monocyte percentages exceeding 10% or falling below 7.4%, coupled with an absolute count under 0.62, face a heightened risk of AECOPD (Lin et al., 2022). Moreover, monocytes have been implicated in the pathogenesis of atherosclerosis, where they act as a primary source of proinflammatory mediators. During atherogenesis, macrophages are drawn to the vessel walls to clear modified low-density lipoproteins (LDLs) and release inflammatory cytokines, thus contributing to the formation of cholesterol-laden plaques. High-density lipoprotein-cholesterol (HDL-C) counteracts these effects by mitigating the proinflammatory and pro-oxidant actions of monocytes. It achieves this by inhibiting macrophage migration, preventing LDL oxidation, and facilitating cholesterol efflux from these cells. Consequently, the monocyte-to-HDL ratio has emerged as a practical indicator for predicting the onset and progression of atherosclerosis, which are critical precursors to cardiovascular events (Ganjali et al., 2018). In our study, the monocytes rose in AMD patients, but how these immune cells take part in the morbidity of AMD and whether abnormal monocytes indicate AMD in some form need further study.

We also obtained 13 relevant miRNAs and 27 TFs by prediction, with three shared TFs. We predicted 17 potentially effective drugs. SLFN11 and GRIN1 shared the drug trichostatin. Trichostatin A (TSA), a potent inhibitor of histone deacetylase (HDAC) effective at nanomolar concentrations, plays a dual role in regulating the eukaryotic cell cycle and reversing morphological transformations in cells (Yoshida and Horinouchi, 1999). Research by Qiang Su and colleagues highlights TSA’s potential to mitigate neuroinflammatory plaque formation and enhance cognitive function. Conditions such as Alzheimer’s disease, anxiety, and depression are closely linked to microglial inflammation, and TSA has been shown to alleviate depressive and anxious behaviors in APP/PS1 mice. Additionally, it reduces CST7 levels in the hippocampus of these mice and in LPS-stimulated BV2 cells (Su et al., 2024). TSA appears to exert its anti-inflammatory effects by increasing the acetylation of non-histone proteins rather than histones, thereby curbing the release of various inflammatory cytokines. This mechanism not only extends survival but also offers protection against acute-on-chronic liver failure (ACLF) in rat models. These findings shed light on how TSA suppresses inflammatory responses in experimental models of autoimmune and inflammatory diseases (Zhang et al., 2015). In addition, some data suggest that TSA through the inhibition of histone deacetylation promotes the apoptotic of the tumor cells. Mukhopadhyay et al. (2006) evaluated TSA as a potential candidate for anticancer therapy in non-small-cell lung cancer. All these reports indicates that TSA has a high probability of inhibiting histone deacetylase and so become a potential drug for AMD, but the definite mechanism need further research.

This study, based on public databases, screened out two biomarkers related to ISR in AMD through a series of bioinformatics analysis methods: SLFN11 and GRIN1. It provides a certain reference value for the pathogenesis, diagnosis, and treatment of AMD. However, our study also has some limitations. On the one hand, the sample size of the existing databases on which the bioinformatics analysis relies is limited. On the other hand, although the potential association of SLFN11 and GRIN1 with ISR was found by bioinformatics methods, the specific mechanism of SLFN11 and GRIN1 in the pathogenesis of AMD remains unclear. In order to further explore this, the sample size should be expanded and AMD patients with different characteristics should be widely collected to build a more comprehensive and representative database. Further *in vitro* and *in vivo* experiments were conducted to investigate the functions of these two genes and their possible mechanisms in AMD. At the same time, a multi-omics integrated analysis method was used to comprehensively analyze their functional networks and explore their upstream and downstream regulatory relationships. In addition, clinical validation studies are carried out to evaluate their value as biomarkers and explore their application potential in personalized treatment to better understand the biological functions and clinical significance of SLFN11 and GRIN1 in AMD and to provide a solid theoretical basis and practical guidance for the diagnosis and treatment of AMD.

## Data Availability

The datasets presented in this study can be found in online repositories. The names of the repository/repositories and accession number(s) can be found in the article/Supplementary Material.
