# Supplementary material for: Identification and validation of integrated stress-response-related genes as biomarkers for age-related macular degeneration
Source: Front Mol Biosci. 2025 Jul 16;12:1583237. doi: 10.3389/fmolb.2025.1583237 (PMC12307159; doi:10.3389/fmolb.2025.1583237)
Supplement: Supplementary file 1 [file Table1.pdf]

Table S1 Information of primers

Primers	Sequences (5’-3’)
SLFN11-F	GGAGTTGCGTTTCTACCCGT
SLFN11-R	GTGTAGCCAGAGTTCCCACC
GRIN1-F	AACCTGCAGCAGTACCATCC
GRIN1-R	GGACCCATCAGTGTCCTTGG
GAPDH-F	CGAAGGTGGAGTCAACGGATTT
GAPDH-R	ATGGGTGGAATCATATTGGAAC
